# A Critical Comparison of Exposure Estimators for Airborne Particulate Matter in Urban Cyclists

**DOI:** 10.3390/toxics14020179

**Published:** 2026-02-17

**Authors:** Elie Al Marj, Ilann Mahou, Roy M. Harrison, Francis D. Pope, Alexandra Fort, Aurelie Charron

**Affiliations:** 1 Unité Mixte de Recherche Epidémiologique et de Surveillance Transports, Travail, Environnement UMRESTTE UMRT9405, Université Gustave Eiffel, Université de Lyon, Université Lyon 1, F-69675 Bron, France; aurelie.charron@univ-eiffel.fr; 2 Laboratoire Ergonomie et Sciences Cognitives pour les Transports LESCOT, Université Gustave Eiffel, Université de Lyon, F-69675 Lyon, France; ilann.mahou@univ-eiffel.fr (I.M.); alexandra.fort@univ-eiffel.fr (A.F.); 3 Division of Environmental Health & Risk Management, School of Geography, Earth & Environmental Sciences, University of Birmingham, Edgbaston, Birmingham B15 2TT, UK; r.m.harrison@bham.ac.uk (R.M.H.); f.pope@bham.ac.uk (F.D.P.)

**Keywords:** individual exposure, traffic-related air pollution, inhaled dose, exposure misclassification, black carbon, ultrafine particles, active commuting

## Abstract

Urban cyclists experience elevated traffic-related air pollutant (TRAP) exposures due to proximity to emissions and increased breathing rates during exercise. Conventional assessments rely on concentration summaries, which may misrepresent actual inhaled doses and misclassify individuals in health studies. Street-level concentrations exhibit high temporal variability, producing non-normal distributions that challenge conventional averaging approaches. This study compares concentration- and dose-based methods to characterize cyclist exposure during urban commuting. Fifty-seven healthy adults completed cycling trips on two 9-km routes (high- and low-traffic) using conventional or electrically assisted bicycles. Real-time monitoring measured black carbon, ultrafine particles, PM_2.5_, and PM_10_. Heart rate-derived breathing rates enabled individualized inhaled dose calculations using three temporal integration methods. Mean concentrations correlated strongly with time-integrated concentrations (r = 0.988–0.998). Simplified dose calculations closely approximated full temporal integration (r > 0.999), with median dose ratios of 0.99–1.01. However, correlations between mean concentrations and inhaled doses were weaker (r = 0.72–0.78). Between 29% and 50% of participants changed exposure quartiles when comparing concentration- and dose-based classifications, with the highest reclassification for ultrafine particles (46–50%). These findings demonstrate that physiological variability substantially influences exposure classification during active commuting, supporting the integration of inhaled dose metrics in cyclist exposure assessment and epidemiological studies.

## 1. Introduction

Outdoor air pollution causes millions of premature deaths annually [1,2], with traffic-related air pollutants (TRAPs) presenting particular health risks due to their proximity to urban populations [3,4,5]. Urban cyclists represent a population of special interest for two reasons. First, cycling populations are increasing in most major cities, driven by public health initiatives promoting active transportation and climate policies targeting net-zero emissions [6,7,8], making the exposure assessment of cyclists increasingly relevant for public health. Second, cyclists experience unique exposure patterns combining close proximity to emissions with elevated and variable breathing rates during physical activity, which can vary 2–3-fold between individuals and bicycle types [5,7,9,10,11,12]. This physiological variability makes cyclists an ideal population for examining the reliability of different exposure assessment approaches.

In recent years, the development of shared-bicycle systems and mobile sensing technologies has enabled a growing number of studies to integrate large-scale cycling trajectory data with real-time pollution monitoring. These approaches allow researchers to identify urban cycling hotspots and assess spatiotemporally dynamic exposure patterns across different routes and time periods. GPS tracking combined with portable air quality sensors has revealed substantial variability in cyclist exposure within urban environments, highlighting the importance of route selection and temporal factors in determining individual pollutant uptake during active commuting [11,12,13,14].

Current exposure assessment methods predominantly rely on concentration-based metrics including arithmetic means, medians, or peak values. These approaches fail to account for two critical factors in active commuting contexts. First, they ignore inter-individual physiological variability in ventilation rates [12]. Second, they inadequately represent the highly skewed concentration distributions characteristic of near-road environments, where brief high-concentration episodes alternate with background levels. Variable breathing rates or minute ventilations (VE) related to exertion do not necessarily correlate with concentration profiles (C), meaning the actual exposure (∫C(t) × VE(t)dt) differs from the product of averages (C × VE), particularly when peaks in concentration and ventilation are temporally misaligned.

Traffic-specific indicators such as ultrafine particles (UFPs) and black carbon (BC) provide more direct markers of vehicle emissions than regulated particulate matters (PM) fractions. UFPs originate primarily from fresh vehicle exhaust [15,16,17] and display steep concentration gradients near roadways [18]. Black carbon, formed through incomplete fossil fuel combustion, similarly concentrates in traffic microenvironments [19,20] and correlates with adverse cardiovascular outcomes [21,22,23]. In contrast, PM_2.5_ and PM_10_ include contributions from multiple sources beyond traffic, reducing their specificity for near-road exposure assessment [24,25,26,27,28].

While several studies have estimated inhaled doses in active commuters [9,10,12,29,30,31,32], systematic comparisons between concentration-based and inhaled dose-based metrics remain limited. Peak-based metrics (95th percentile and maximum) have occasionally been determined to capture high-exposure events [33,34], though their use is less common than mean-based approaches.

This study addresses these gaps by evaluating the proportionality between various exposure metrics using real-world cycling data on contrasting urban routes, quantifying potential exposure misclassification, and providing evidence-based recommendations for metric selection in traffic pollution research.

## 2. Materials and Methods

### 2.1. Study Design and Participants

Fifty-seven healthy adults aged 18–40 years were recruited through university mailing lists and local cycling groups. The sample consisted of 31 women and 26 men, distributed as shown in Supplementary Table S1. Inclusion criteria required regular cycling experience. Exclusion criteria included cardiovascular or respiratory disorders and cardiovascular medication use. Informed consent was obtained from all subjects involved in the study. The institutional ethics committee approved all procedures. Of the 57 recruited participants, 48 provided complete data.

### 2.2. Study Location and Cycling Routes

Experiments were conducted in Bron, France (45°44′22″ N, 4°54′50″ E), within the Lyon metropolitan area. Two contrasting 9-km cycling routes (comparable in elevation profile and surface quality) were selected to maximize traffic exposure differences while maintaining representativeness for typical urban commuting scenarios. The close-to-traffic route (CTF) followed major arterial roads, including Franklin Roosevelt Avenue, with high traffic volumes, frequent signalized intersections, commercial activities, and bus transit corridors. This route represents typical commuting pathways for urban cyclists. The away-from-traffic route (ATF) traced residential neighborhoods, dedicated cycling paths through a business park (Parc du Chêne), and green corridors with less motorized traffic, reflecting alternative route choices available to cyclists seeking lower-exposure pathways. Both routes originated from the Gustave Eiffel University campus (Cité des Mobilités), allowing standardized departure procedures and equipment preparation, with route characteristics reflecting typical urban cycling environments in medium-sized European metropolitan areas. The 9-km route length corresponds to typical urban cycling patterns, as recent French national mobility surveys indicate that trips exceeding 10 km represent only 13% of daily movements, with over 80% of longer distances covered by car rather than bicycle [35].

### 2.3. Experimental Protocol and Data Collection

Data collection occurred during autumn (September–December 2024) and spring (March–June 2025). Each participant completed both routes within 48 h using randomly assigned conventional bicycles (CB) or electrically-assisted bicycles (EAB). The same bicycles dedicated to the project were used to standardize equipment. Sessions occurred during weekday rush hours (08:00–09:00 and 17:00–18:00) to be representative of commuting traffic conditions. Each ride lasted approximately 40–45 min, and participants were instructed to strictly follow the experimenter and to maintain a steady cycling pace with him. Temperature and humidity were recorded on-site during each session. During autumn, mean temperatures were 13.1 °C (±5.2 °C) and 13.8 °C (±4.4 °C) during the CTF and ATF field campaigns, respectively. In spring, mean temperatures increased to 21.4 °C (±5.4 °C) and 20.5 °C (±4.8 °C) for the same two field campaigns respectively. Relative humidity was higher in autumn at 69.0% (±8.4%) and 67.6% (±9.6%) for the CTF and ATF field campaigns respectively, compared to spring values of 46.9% (±14.3%) and 48.6% (±12.3%). Meteorological data from the Météo-France station located at Bron Airport (one kilometer away) showed minimal precipitation during both campaigns, with autumn recording 2.7 ± 6.4 mm and spring recording 2.5 ± 6.2 mm, with no precipitation occurring during experimental sessions. Sessions were postponed during extreme temperatures (>35 °C) and strong winds (>40 km/h). These meteorological parameters were monitored to account for their potential influence on pollutant concentrations through atmospheric mixing and dilution effects. The standardized cycling pace protocol ensures systematic comparison between exposure assessment methods by minimizing inter-individual differences in physical exertion. However, this controlled approach may not fully capture the natural breathing rate variability that occurs during spontaneous cycling, where cyclists adjust speed based on traffic conditions, personal fitness, and route familiarity.

### 2.4. Personal Exposure Monitoring

#### 2.4.1. Particulate Matter Measurements

Particulate matter was measured with a portable optical particle counter (Aerocet 532, Met One Instruments, Grants Pass, OR, USA) operating at 2.83 L/min flow rate. The device reports mass concentrations (PM_1_, PM_2.5_, PM_4_, PM_7_, PM_10_, and total suspended particles) and particle counts across eight size bins (0.3 and 10 µm). Data were recorded at 1-min intervals with ±10% accuracy (0–1000 µg/m^3^). Performance has been validated against gravimetric reference methods [34,36]. Weekly zero checks with HEPA filters and monthly flow calibrations ensured reliability.

#### 2.4.2. Ultrafine Particle Assessment

Ultrafine particle number concentrations were assessed using a diffusion size classifier (DiSCmini, Testo SE & Co. KGaA, Titisee-Neustadt, Germany) reporting the total number of concentrations (10^3^ to 10^6^ particles/cm^3^), mean diameter (10–300 nm), and lung-deposited surface area (LDSA) fraction. Measurements were logged at one-second resolution with an accuracy of ±30%. Zero tests with HEPA-filtered air before and after sessions controlled drift. The DiSCmini has been validated against reference instruments such as condensation particle counters (CPC) and scanning mobility particle sizers (SMPS) [37,38,39].

#### 2.4.3. Black Carbon Monitoring

Equivalent black carbon (eBC) was measured with a micro-aethalometer (MA200, AethLabs, San Francisco, CA, USA) recording light attenuation at five wavelengths (375–880 nm) at 10 s intervals and a 150 mL/min flow rate. The instrument uses a mass absorption cross-section (MAC) of 10.12 m^2^/g at 880 nm to convert optical attenuation to eBC mass concentration (manufacturer specification). The dual-spot correction method reduced filter-loading artifacts [19,20]. To improve data quality when sampling interval times below or equal to 10 s are used, raw signals were corrected using optimized noise averaging (ONA), which effectively minimizes false negative values [19,40]. The ONA algorithm set an incremental light attenuation (ΔATN) threshold to force the algorithm to calculate a BC concentration only when there is enough black carbon on the filter to detect a variation of light attenuation (ATN) value. The ΔATN threshold needs to be defined, and the best choice depends on the type of measurements. Different ΔATN thresholds were compared in this study. As for the observations of [19] for mobile measurements, ΔATN thresholds higher than 0.02 result in a curve that is too smoothed to capture all the observed concentration variations. However, unlike them, a ΔATN threshold of 0.01 leads to noise and negative values. We concluded that a ΔATN threshold of 0.02 preserves temporal variations in BC concentrations relevant to mobile monitoring conditions while eliminating virtually all negative values. Strong agreement with reference aethalometers has been demonstrated [41,42,43,44].

#### 2.4.4. Physiological Monitoring

The heart rate of the participants was continuously recorded at 1-s intervals using GPS-enabled sports watches (Garmin Forerunner 255, Garmin Ltd., Olathe, KS, USA). This device also logged geospatial coordinates, allowing for the alignment of physiological and environmental data.

#### 2.4.5. Minute Ventilation Rate Estimation

Minute ventilation (VE, L/min) was estimated from heart rate (HR) using sex-specific exponential equations validated against facemask measurements [12]. For men, the relationship was expressed as VE = exp(1.16 + 0.021 × HR), and for women as VE = exp(0.99 + 0.021 × HR). This method provides a practical approximation of ventilation in field settings, although inter-individual variability in metabolic response may not be fully captured.

### 2.5. Exposure Estimation Approaches

Five concentration-based estimators were calculated: arithmetic mean, median, maximum, 95th percentile (P95), and time-integrated concentration. The latter is calculated as: ∫C(t)dt = Σ(Ci × Δt), representing the area under the concentration-time curve. Mean concentrations are most commonly used in exposure assessment [45,46]. Medians are assumed to characterize typical exposures while reducing extreme value influence. Peak metrics (P95, maximum) capture high-exposure events in traffic microenvironments [47]. Time-integrated concentration (Equation: ∫C(t)dt = Σ(Ci × Δt)) represents a methodological approach explicitly used in this study to fully account for temporal variability in exposure profiles. It serves as a comparator to conventional summary metrics.

Inhaled dose is the product of pollutant concentration, ventilation, and exposure duration [9,10,12,31,32]. Three dose calculation approaches were compared. Approach 1, designated as the reference method, calculated dose by summing products of instantaneous (10-s) concentration and ventilation measurements (1-min) across the entire commute: Dose1 = Σ(Ci × VEi × Δt), where Δt = 10/60 min. This approach preserves full temporal coupling between dynamically fluctuating concentrations and ventilation rates, allowing individualized dose estimation while requiring complete time-series data. Approach 2 simplified calculations by using mean concentration across the entire commute multiplied by cumulative ventilation volume: Dose2 = Cmean × Σ(VEi × Δt). This method maintains individual ventilation profiles but assumes constant concentration exposure, reducing computational requirements while retaining sensitivity to inter-individual physiological differences. This approach represents cases in which concentrations are only known for the entire duration of the journey (e.g., sampling with a personal impactor). Approach 3 relied on time-integrated concentration exposure multiplied by mean ventilation rate: Dose3 = [Σ(Ci × Δt)] × VEmean. This approach captures temporal concentration variability but assumes constant ventilation, offering an alternative simplification with distinct assumptions about dominant sources of exposure variance. All dose calculations applied appropriate unit conversions resulting in mass-based doses (micrograms) for particulate matter and black carbon, or count-based doses (total particle numbers) for ultrafine particles. In this study, inhaled dose is defined as the pollutant quantity taken into the respiratory system through mouth or nose, excluding losses from exhalation and clearance. More precise dose metrics account for particle deposition and clearance but require advanced modeling beyond this field investigation [12,48,49].

### 2.6. Statistical Analysis Framework

Route differences were assessed using Wilcoxon rank-sum tests. Proportionality between methods was evaluated using Pearson correlations, median dose ratios, and interquartile coefficient of dispersion (IQCD = [(Q3 − Q1)/(Q3 + Q1)] × 100%). Systematic bias was tested using Wilcoxon signed-rank tests comparing dose ratios to unity. Exposure ranking stability was assessed through quartile classification changes. Peak exposure days were defined as sessions with ≥15% of measurements exceeding the 90th percentile. Statistical significance was set at α = 0.05. The normality of pollutant variables was assessed using the Shapiro–Wilk test, complemented by skewness and kurtosis estimates (Supplementary Table S2). Analyses used R version 4.4.0 (R Core Team, 2024).

## 3. Results

### 3.1. Descriptive Analysis of Physiological Parameters and Pollutant Exposure Levels

#### 3.1.1. Physiological Basis for Dose Estimation

Heart rate responses differed significantly by bicycle type. On CTF routes, CB users averaged 115.9 ± 21.3 beats per minute (bpm) compared with 94.6 ± 16.4 bpm for EAB users (*p* < 0.001). On ATF routes, CB users averaged 117.3 ± 20.9 bpm versus 90.9 ± 13.9 bpm for EAB users (*p* < 0.001). Estimated minute ventilation mirrored these heart rate differences. Using the sex-specific HR-VE equations, ventilation was higher for CB users than EAB users on both routes. VE averaged 35.4 ± 13.4 L/min for males and 37.2 ± 24.4 L/min for females riding CB, versus 23.9 ± 8.6 L/min (males) and 21.5 ± 8.4 L/min (females) for EAB users.

#### 3.1.2. Route-Based Concentration Differences

Pollutant concentrations differed substantially between routes (Table 1). Black carbon showed the largest route differential, with CTF concentrations reaching 2.45 times those observed on ATF routes (CTF median 2.33 μg/m^3^ [IQR 1.58–3.88] versus ATF median 1.01 μg/m^3^ [IQR 0.72–1.59], *p* < 0.001). PM_10_ exhibited the second-largest separation, with concentrations on CTF routes exceeding ATF values by 2.18-fold (CTF median 43.90 μg/m^3^ [IQR 33.83–62.90] versus ATF median 32.30 μg/m^3^ [IQR 22.25–42.48], *p* < 0.001). PM_10_ concentrations within traffic environments contain a higher proportion of coarse particles from resuspension and non-exhaust emissions compared to background locations, which likely contributes to the observed 2.18-fold difference between routes. Ultrafine particle number concentrations were 1.89 times higher on CTF routes (CTF median 10,023 particles/cm^3^ [IQR 6013–16,569] versus ATF median 6385 particles/cm^3^ [IQR 4424–10,930], *p* < 0.001). As expected, fine particulate matter (PM_2.5_) displayed the smallest and only non-significant route differential (ratio 1.15, CTF median 13.75 μg/m^3^ [IQR 8.10–19.65] versus ATF median 8.80 μg/m^3^ [IQR 6.22–20.45], *p* = 0.167).

**Figure 1:**
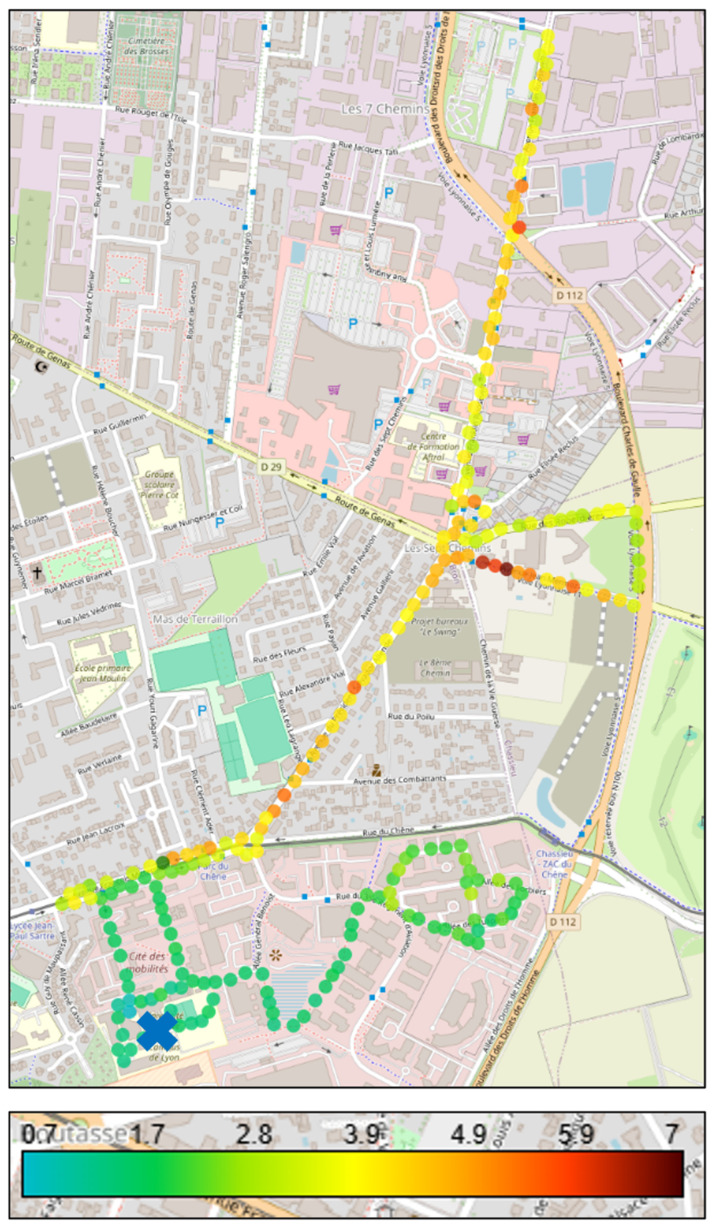
Spatial distribution of real-time BC concentrations (µg/m^3^) measured during cycling experiments in Bron (Lyon metropolitan area, France). The map shows the location of the experimental site (Gustave Eiffel University campus, Cité des Mobilités), indicated by the blue X, which served as both departure and return point. The two cycling routes are displayed with distinct gradients: the CTF route (red-orange gradient), primarily along major traffic corridors (e.g., François Mitterrand Avenue, Franklin Roosevelt Avenue, Route de Genas), and the ATF route (green gradient), located mainly on lower-traffic streets and residential areas. Main road infrastructure is visible in the background map to contextualize traffic influence. Colored markers represent mean BC concentrations averaged across all experimental sessions, illustrating the spatial contrast in exposure between different traffic environments.

These route-based exposure differences are illustrated in Figure 1, which displays real-time BC measurements across both experimental pathways, clearly demonstrating the 2.45-fold concentration difference between CTF and ATF routes.

#### 3.1.3. Route-Based Inhaled Dose Differences

Inhaled doses mirrored concentration patterns (Table 2). Using Approach 1, close-to-traffic doses exceeded away-from-traffic doses with ratios of 2.98 for black carbon (CTF median 3.37 μg [IQR 2.11–6.19] versus ATF median 1.26 μg [IQR 0.77–2.29], *p* < 0.001), 2.20 for PM_10_ (CTF median 66.33 μg [IQR 37.98–100.93] versus ATF median 36.46 μg [IQR 22.22–64.31], *p* = 0.001), 2.07 for ultrafine particles (CTF median 1.65 × 10^10^ particles [IQR 0.98 × 10^10^–3.05 × 10^10^] versus ATF median 0.93 × 10^10^ particles [IQR 0.50 × 10^10^–1.36 × 10^10^], *p* < 0.001), and 1.21 for PM_2.5_ (CTF median 14.68 μg [IQR 9.28–28.41] versus ATF median 12.46 μg [IQR 6.63–20.09], *p* = 0.151). These route-based dose differences remained consistent across all three estimation approaches, indicating robust differentiation regardless of temporal integration strategy. Approach 2 (mean concentration method) demonstrated CTF-to-ATF ratios of 2.95 for BC, 2.24 for PM_10_, 2.04 for UFP, and 1.21 for PM_2.5_. Approach 3 (integrated concentration method) produced ratios of 2.96, 2.25, 2.04, and 1.21 for the same pollutants, respectively.

### 3.2. Comparison of Exposure Assessment Approaches

#### 3.2.1. Different Dose Calculation Approaches

Simplified dose approaches showed excellent agreement with full temporal integration (Table 3). Approach 2 (mean concentration method) demonstrated near-perfect correlation with Approach 1 (r > 0.999) with minimal bias (<2%) across all pollutants. Approach 3 (mean ventilation method) showed slightly larger but consistent positive bias (2–5%) while maintaining high correlation.

Similar agreement between dose calculation approaches was observed for the ATF routes (Supplementary Table S3), with slightly lower IQCD values (0.3–1.7%) reflecting the more stable concentration conditions in low-traffic environments.

Among the 48 participants, 13 participant-route combinations (27%) exhibited slightly poorer proportionality between approaches (ratio <0.9 or >1.1). These outliers had elevated heart rates (113.3 ± 18.0 vs. 103.2 ± 19.7 bpm, *p* = 0.048) but no differences in ventilation variability, concentration variability, or peak exposure frequency (all *p* > 0.05), suggesting random temporal alignment variations rather than systematic factors.

#### 3.2.2. Proportionality Analysis

Estimates of ventilation rates are not always available (e.g., studies comparing exposure at the street level regardless of individual physiology). Hence, concentration estimators were compared to both the time-integrated concentrations, which fully take into account concentration peaks, and the ventilation-adjusted inhaled dose (Approach 1 only).

##### Concentration Estimators Versus Time-Integrated Concentration

On CTF routes, mean concentrations showed high proportionality with full time-integrated concentrations (Pearson r = 0.988–0.998; IQCD of 4.5–4.6%). Median and P95 performed moderately (r = 0.881–0.992; IQCD 6.8–16.0% and r = 0.895–0.980; IQCD of 9.7–15.5%). Maximum concentrations showed poor agreement with time-integrated concentrations, exhibiting the weakest correlation (r = 0.402–0.684; IQCD 25–48.4%) (Table 4).

Concentration estimators showed comparable performance on the ATF routes (Supplementary Table S4), with mean concentrations maintaining high correlation with time-integrated values (r = 0.989–0.995).

##### Concentration Estimators Versus Ventilation-Adjusted Dose (Inhaled Dose)

With ventilation included, proportionality decreased substantially. Mean concentration retained the highest correlation with dose (r = 0.722–0.775) and showed an acceptable ratio variability (IQCD of 24.2–26.3%). Again, median and P95 produced intermediate performance, while maximum concentration correlated the poorest with dose (r = 0.354–0.694; IQCD up to 55%) (Table 5).

**Figure 2:**
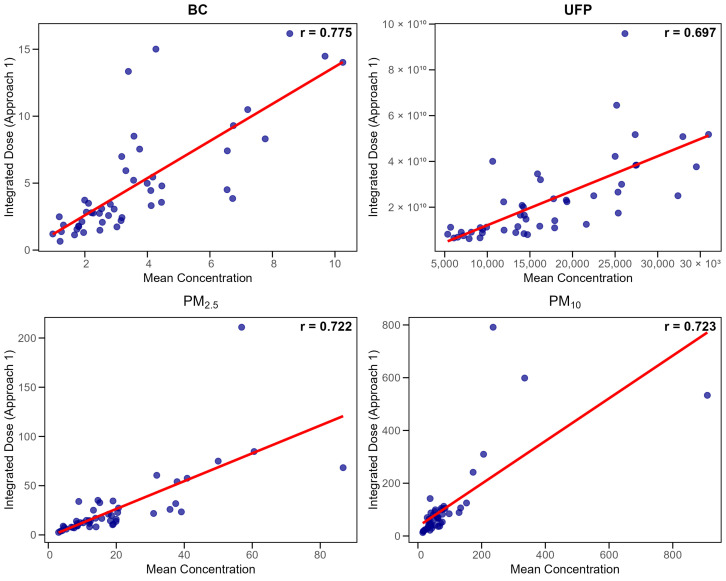
Scatter plots showing correlations between mean concentrations and integrated doses (Approach 1) for each pollutant. Individual data points represent participant measurements, with red regression lines indicating overall trends.

Figure 2 demonstrates the relationship between mean concentrations and integrated doses for each pollutant (Approach 1). Despite moderate correlations between concentration and dose metrics (r = 0.72–0.78), participants experiencing similar pollutant concentrations had different actual uptake due to physiological variability during cycling.

Lower correlations between concentration metrics and inhaled dose were observed for the ATF routes (Supplementary Table S5, r = 0.46–0.74 for means), though IQCD values remained similar (23–25%), indicating that physiological variability affects dose estimation regardless of traffic exposure level.

#### 3.2.3. Exposure Ranking Stability

Between 29–50% of participants changed exposure quartiles when comparing concentration-based versus dose-based classifications (Table 6). UFP showed the highest reclassification rate (46–50%), while BC showed the lowest (29–39%). Major misclassifications (≥2 quartile changes) remained rare, affecting only 2–8% of participants.

**Figure 3:**
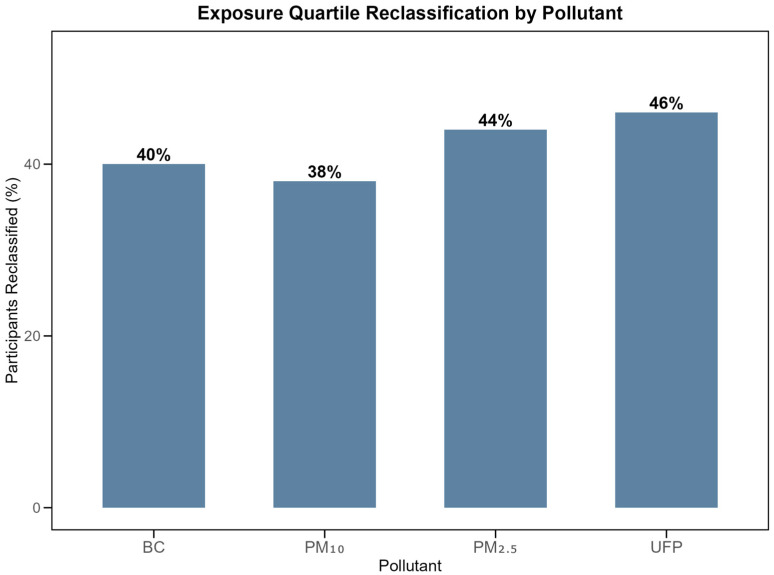
Percentage of participants changing exposure quartiles when ranked by integrated dose versus mean concentration. UFP showed the highest reclassification rate (46%).

This quartile reclassification pattern is illustrated in Figure 3, demonstrating the magnitude of exposure misclassification when physiological factors are included in the exposure estimation.

## 4. Discussion

The proportionality analysis demonstrates systematic divergence between concentration-based and dose-based exposure classifications across all examined pollutants, with fundamental implications for environmental epidemiology methodology [12,46].

The near-perfect correlations between the three dose calculation methods (r > 0.999) validate simplified approaches for practical implementation for the conditions of our study (i.e., urban cycling at low speeds, traffic emission exposure, and urban background conditions). This robustness supports the feasibility of dose-based assessments in large-scale studies where computational efficiency matters [9,12]. Furthermore, the results indicate that satisfactory dose-exposure estimates can be derived using less temporally-resolved instruments or filter-based sampling with laboratory analysis, expanding methodological options for resource-limited studies. These strong correlations were observed under the relatively homogeneous conditions evaluated here, i.e., urban cycling routes with moderate elevation changes. Greater divergence between methods could occur in environments with highly heterogeneous pollution sources (e.g., industrial zones mixed with traffic) or variable terrain and topography inducing irregular breathing patterns (e.g., steep hills), where concentration peaks and elevated breathing rates occur at different times, potentially affecting dose estimates more substantially. The analysis of outlier participants (27% with ratios outside 0.9–1.1) revealed elevated heart rates but no systematic differences in other characteristics, suggesting that even in our controlled conditions, random temporal misalignment between concentration peaks and ventilation patterns can occur.

Among concentration estimators, arithmetic means demonstrated the best performance, showing excellent agreement with time-integrated concentrations (r = 0.988–0.998). This performance can be explained by the sensitivity of mean to high values, effectively capturing concentration peaks characteristic of near-road environments. However, mean concentrations showed moderate correlation with inhaled doses (r = 0.72–0.78), with 24–26% variability in dose-concentration ratios. This substantial individual variability indicates that participants with identical measured concentrations had markedly different actual pollutant uptake due to differences in ventilation rates, potentially introducing substantial bias in individual-level health studies. The 29–50% participant reclassification rate when incorporating physiological factors quantifies the exposure misclassification inherent in concentration-only approaches for studies involving participants. This finding is particularly significant given that exposure misclassification typically attenuates exposure–response relationships, masking true health associations or introducing bias in effect estimation [50,51].

The pollutant-specific route contrasts reveal important considerations for exposure assessment. Black carbon and UFPs showed strong spatial discrimination (2.5-fold and 1.9-fold differences), confirming their value as traffic-specific markers reflecting primary emissions and near-source gradients [45,46,51]. The minimal route discrimination of PM_2.5_ (1.15-fold) reflects substantial regional background contributions from secondary aerosols and non-traffic sources, diminishing its utility for characterizing near-road exposure gradients [22,23,48,49]. The intermediate behavior of PM_10_ (2.18-fold) reflects mixed contributions from traffic-generated coarse particles including resuspension, and non-exhaust emissions [26].

Despite source differences, concentration–dose correlations were remarkably similar across pollutants (r = 0.70–0.78 for means), indicating physiological variability affects exposure assessment regardless of pollutant characteristics. This suggests metric selection should prioritize study objectives over pollutant type. While traffic-specific indicators (BC, UFP) excel at identifying traffic sources and distinguishing high- from low-traffic environments, they do not provide better individual dose estimates than PM fractions when physiological variability is considered, as all pollutants showed similar concentration-dose correlations (r = 0.70–0.78).

These findings align with recent studies emphasizing the importance of dose-based over concentration-based exposure assessment. Zuurbier et al. [5] demonstrated that cyclists show approximately two-fold higher minute ventilation compared to motor vehicle occupants, leading to nearly twice the pollution doses despite similar concentration exposures. This physiological difference highlights why concentration metrics alone may be insufficient for active populations.

Critically, Velasco et al. [52] showed that using exposure concentration instead of inhaled dose can cause biased interpretation of health risks associated with different transport modes, recommending that assessments should focus on inhaled dose rather than exposure concentrations. This recommendation is particularly relevant for active commuting where physical effort varies considerably. As Adams et al. [53] noted, dose-based exposure approaches account for individual inhalation rates and changes due to energy expenditure variations.

As highlighted by Borghi et al. [10], current exposure assessment approaches typically focus on external concentrations rather than actual inhaled amounts, potentially leading to misinterpretation of health risks across different transport modes. Vouitsis et al. [54] provided quantitative evidence, reporting that cyclists inhaled 35% more particulate matter and 62% more black carbon compared to bus passengers. Dons et al. [12] further demonstrated the complexity of ventilation estimation, finding that while physical activity categories can predict ventilation over longer periods, substantial deviations occur during specific activities, with continuous measures better reflecting individual variation.

Our observed 29–50% participant reclassification when incorporating physiological factors directly supports these concerns about exposure misclassification in active populations. These methodological considerations have particular relevance for vulnerable road users, including delivery workers, recreational cyclists, and pedestrians, who may experience systematic exposure underestimation when assessments rely solely on concentration measurements.

## 5. Implications for Study Design

Individual-level investigations, particularly case-control studies examining personal exposure gradients, will benefit from dose-based approaches given the substantial participant reordering observed. The 46–50% reclassification rate for UFPs is particularly concerning given their emerging importance in health studies [4,15]. Since computing time-integrated concentrations do not seem to provide any significant improvement compared to the arithmetic mean, population-level studies comparing groups or areas may reasonably use arithmetic means, as individual differences average out while preserving spatial contrasts [50,51,55,56]. As well, infrastructure assessment and public health decision-making can rely on arithmetic mean concentrations as reasonable exposure proxies when comparing routes or interventions.

Beyond research applications, these findings have direct implications for practical exposure assessment and urban planning. Municipal authorities evaluating cycling infrastructure effectiveness should consider that physically demanding routes (steep terrain) and high-traffic areas increase both pollutant concentrations and breathing rates [5,11], leading to higher pollutant intake than concentration measurements alone would indicate. Practical applications include developing cycling navigation applications that combine air quality data with route characteristics, and issuing enhanced pollution warnings specifically for cyclists and pedestrians during high pollution episodes, acknowledging their intake rates are 2–3 times higher than resting levels [5,11]. Urban planners should prioritize protected bike lanes on busy streets where active commuters have limited route alternatives. For individual cyclists, the simplified dose calculation methods validated here (r > 0.999 agreement with full temporal integration) demonstrate that including physiological factors requires only simple calculations. This opens possibilities for smartphone applications that combine heart rate data from standard fitness trackers with real-time pollution information to provide personalized exposure estimates.

## 6. Limitations

This study has limitations that should be considered when interpreting the results. Ventilation estimates relied on heart rate equations [12,31,32] validated in similar populations but not independently verified through direct spirometry or facemask measurements [12], introducing uncertainty in dose calculations. The doses represent inhaled rather than deposited quantities, as deposition and clearance modeling was beyond the scope of this study [48,49]. Our conclusions are based on comparison with an inhaled dose that itself contains uncertainties from HR-based ventilation estimation and do not account for health outcome relationships, which could provide alternative validation of exposure metrics. The healthy young adult population may underestimate variability in vulnerable groups with greater physiological heterogeneity. The controlled route design, while ensuring systematic contrasts, may not capture all real-world commuting variability in route selection, timing, and intensity [57,58]. Maximum concentrations showed consistently poor performance across all analyses, confirming their inadequacy for cumulative exposure assessment during active commuting [12,31,32,50].

## 7. Conclusions

Urban cyclists experience elevated traffic-related air pollutant exposures through both proximity to emissions and increased ventilation rates. This investigation demonstrates that physiological integration substantially alters exposure classification compared to concentration-only approaches, with 29–50% of participants changing exposure categories when including breathing rates.

Public health implications are significant. Ignoring physiological variability in exposure assessment can lead to systematic underestimation of true pollutant uptake, particularly in physically active populations where ventilation rates substantially exceed resting levels during physical activity. This misclassification weakens epidemiological evidence linking traffic pollution to health outcomes and may delay implementation of protective measures for vulnerable road users, including cyclists, pedestrians, and outdoor workers. Emphasizing inhaled dose estimation is essential for both individual-level health assessments and population-based public health studies involving physically active populations.

Individual-level health investigations should prioritize dose-based metrics when feasible, while population-level assessments may reasonably rely on concentration measurements for route or infrastructure comparisons. The validated simplified dose calculation approaches presented here (r > 0.999 agreement) provide practical pathways for incorporating physiological factors without requiring extensive computational resources.

Future research should extend dose modeling to include particle deposition and clearance mechanisms, validate approaches across diverse populations including vulnerable groups, and quantify how exposure metric selection influences health outcome associations. Including physiological monitoring in active transportation research represents a critical step toward reducing exposure misclassification and strengthening the scientific foundation for traffic pollution health policy.

## Figures and Tables

**Table 1:** Route-based concentration differences.

Pollutant	N_CTF_	CTF Mean ± SD	CTF Median [IQR]	N_ATF_	ATF Mean ± SD	ATF Median [IQR]	Ratio (CTF/ATF)	*p*-Value
PM_2.5_ (μg/m^3^)	48	19.930 ± 17.060	13.750 [8.100–19.650]	48	17.340 ± 16.950	8.800 [6.220–20.450]	1.150	0.167
PM_10_ (μg/m^3^)	48	88.370 ± 135.590	43.900 [33.830–62.900]	48	40.500 ± 25.850	32.300 [22.250–42.480]	2.180	<0.001
BC (μg/m^3^)	48	3.660 ± 2.270	2.330 [1.580–3.880]	48	1.500 ± 1.200	1.010 [0.720–1.590]	2.450	<0.001
UFP (×10^4^ particles/cm^3^)	48	1.700 ± 0.840	1.000 [0.600–1.660]	48	0.900 ± 0.530	0.640 [0.440–1.090]	1.890	<0.001

Note: CTF = Close-to-Traffic; ATF = Away-from-Traffic. Values shown as mean ± standard deviation and median [interquartile range]. Ratio = CTF/ATF. *p*-values from Wilcoxon rank-sum tests.

**Table 2:** Route-based inhaled dose differences.

Pollutant	CTF Mean ± SD	CTF Median [IQR]	ATF Mean ± SD	ATF Median [IQR]	Ratio (CTF/ATF)	*p*-Value
PM_2.5_ (μg)	26.250 ± 33.370	14.680 [9.280–28.410]	21.620 ± 30.860	12.460 [6.630–20.090]	1.210	0.151
PM_10_ (μg)	107.600 ± 151.800	66.330 [37.980–100.930]	48.900 ± 43.300	36.460 [22.220–64.310]	2.200	0.001
BC (μg)	4.900 ± 4.070	3.370 [2.110–6.190]	1.650 ± 1.210	1.260 [0.770–2.290]	2.980	<0.001
UFP (×10^10^ particles)	2.270 ± 1.810	1.650 [0.980–3.050]	1.100 ± 1.140	0.930 [0.500–1.360]	2.070	<0.001

Note: CTF = Close-to-Traffic; ATF = Away-from-Traffic. Values shown as mean ± standard deviation and median [interquartile range]. Ratio = CTF/ATF. *p*-values from Wilcoxon rank-sum tests.

**Table 3:** Performance of simplified dose estimation approaches compared to instantaneous integration (Approach 1) (CTF routes) (N = 48, per pollutant).

Pollutant	Method	Correlation (r)	Median Ratio	IQCD%
PM_2.5_	Approach 2	1.000	1.000	0.600
	Approach 3	1.000	1.030	1.000
PM_10_	Approach 2	0.999	1.010	1.500
	Approach 3	0.999	1.040	2.400
BC	Approach 2	0.999	0.990	1.600
	Approach 3	0.999	1.010	1.400
UFP	Approach 2	0.999	0.990	1.900
	Approach 3	0.999	1.020	2.000

Note: All comparisons against Approach 1 (full temporal integration). Approach 2 = mean concentration × total ventilation; Approach 3 = integrated concentration × mean ventilation. r = Pearson correlation; IQCD = Interquartile Coefficient of Dispersion of dose ratios.

**Table 4:** Performance of concentration-based estimators (mean, median, P95, max) versus time-integrated concentration (CTF routes).

Pollutant	Concentration Method	N	r	IQCD%
PM_2.5_	Mean	48	0.995	4.500
	Median	48	0.992	6.800
	P95	48	0.959	9.700
	Max	48	0.579	25.000
PM_10_	Mean	48	0.998	4.500
	Median	48	0.989	9.500
	P95	48	0.980	12.200
	Max	48	0.619	47.700
BC	Mean	48	0.993	4.600
	Median	48	0.947	13.100
	P95	48	0.929	15.500
	Max	48	0.684	33.700
UFP	Mean	48	0.988	4.500
	Median	48	0.881	16.000
	P95	48	0.895	10.200
	Max	48	0.402	48.400

Note: r = Pearson correlation coefficient; IQCD = Interquartile Coefficient of Dispersion.

**Table 5:** Performance of concentration-based estimators (mean, median, P95, max) versus inhaled dose (approach 1) (CTF routes).

Pollutant	Concentration Method	N	r	IQCD%
PM_2.5_	Mean	48	0.722	24.800
	Median	48	0.709	21.500
	P95	48	0.800	27.500
	Max	48	0.441	30.800
PM_10_	Mean	48	0.723	24.200
	Median	48	0.698	25.300
	P95	48	0.762	22.100
	Max	48	0.694	55.000
BC	Mean	48	0.775	25.200
	Median	48	0.753	31.900
	P95	48	0.696	31.400
	Max	48	0.486	39.900
UFP	Mean	48	0.697	26.300
	Median	48	0.620	32.900
	P95	48	0.620	30.900
	Max	48	0.354	54.300

Note: r = Pearson correlation coefficient; IQCD = Interquartile Coefficient of Dispersion.

**Table 6:** Exposure ranking stability across estimation approaches.

Pollutant	Route	N	Quartile Changes (%)	Major Changes n (%)
PM_2.5_	CTF	48	43.8	1 (2.1%)
	ATF	48	35.4	1 (2.1%)
PM_10_	CTF	48	37.5	4 (8.3%)
	ATF	48	47.9	3 (6.2%)
BC	CTF	48	39.6	1 (2.1%)
	ATF	48	29.2	3 (6.2%)
UFP	CTF	48	45.8	2 (4.2%)
	ATF	48	50.0	2 (4.2%)

Note: Quartile Changes = percentage of participants changing exposure quartile. Major Changes = number of participants changing ≥2 quartiles.

## Data Availability

Data presented in the study are included in the paper and Supplementary Materials. Additional needs can be directed to the corresponding author.

## Supplementary Materials

The following supporting information can be downloaded at: https://www.mdpi.com/article/10.3390/toxics14020179/s1, Table S1. Participant distribution by bicycle type and sex; Table S2. Normality assessment of pollutants variables; Table S3. Performance of simplified dose estimation approaches compared to instantaneous integration (Approach 1) (ATF routes) (N = 48, per pollutant); Table S4. Performance of concentration-based estimators (mean, median, P95, max) versus time-integrated concentration (ATF routes); Table S5. Performance of concentration-based estimators (mean, median, P95, max) versus inhaled dose (approach 1) (ATF routes).

## Abbreviations

The following abbreviations are used in this manuscript:

ATF	Away-from-traffic routes
ATN	Light attenuation value
BC	Black carbon
C	Concentration
CB	Conventional bicycles
CPC	Condensation particle counter
CTF	Close-to-traffic routes
ΔATN	Incremental light attenuation
EAB	Electrically-assisted bicycles
eBC	Equivalent black carbon
HR	Heart rate
LDSA	Lung-deposited surface area
MAC	Mass absorption cross-section
ONA	Optimized noise averaging
PM	Particulate matter
SMPS	Scanning mobility particle sizer
TRAP	Traffic-related air pollutant
UFP	Ultrafine particle
VE	Breathing rate or minute ventilation
