# Supplementary material for: A Critical Comparison of Exposure Estimators for Airborne Particulate Matter in Urban Cyclists
Source: Toxics. 2026 Feb 17;14(2):179. doi: 10.3390/toxics14020179 (PMC12945186; doi:10.3390/toxics14020179)
Supplement: Supplementary file 1 [file toxics-14-00179-s001.zip › toxics-4151379-supplementary.pdf]

Supplementary Material

A Critical Comparison of Exposure Estimators for Airborne Particulate Matter in Urban Cyclists.

Elie Al Marj[1]*, Ilann Mahou[2], Roy M. Harrison[3], Francis D. Pope[3], Alexandra Fort[2], Aurelie Charron[1]

1 UMRESTTE UMRT9405, Université Gustave Eiffel, Université de Lyon, Université Lyon 1F-69675, Bron, France.
2 LESCOT, Univ Gustave Eiffel, Univ Lyon, F-69675, Lyon, France.
3 Division of Environmental Health & Risk Management, School of Geography, Earth & Environmental Sciences, University of Birmingham, Edgbaston, Birmingham, B15 2TT, United Kingdom.
* Correspondence: elie.almarj@univ-eiffel.fr

Table S1. Participant distribution by bicycle type and sex.

Bicycle type	Women	Men	Total
Conventional (CB)	17	12	29
Electrically-assisted (EAB)	14	14	28
Total	31	26	57

Table S2. Normality assessment of pollutants variables.

Route	Pollutant	N	Shapiro p	Skewness	Kurtosis	Normality Assessment
ATF	$PM_{2.5}$	48	1.6671E-07	1.769	5.405	No
	PM_{10}	48	2.30971E-06	1.969	7.543	No
	BC	48	2.067E-06	1.554	4.658	No
	UFP	48	8.960E-05	1.278	4.499	No
CTF	$PM_{2.5}$	48	9.596E-07	1.882	6.806	No
	PM_{10}	48	2.980E-12	4.901	29.395	No
	BC	48	3.594E-05	1.285	3.932	No
	UFP	48	0.014	0.552	2.324	No

Note: Normality was assessed for each pollutant and for each trip using the same statistical tests. Results were consistent across pollutants and trips; therefore, only the aggregated results are reported here. Detailed results by pollutant and by trip can be provided upon request.

Table S3. Performance of simplified dose estimation approaches compared to instantaneous integration (Approach 1) (ATF routes) (N=48, per pollutant).

Pollutant	Method	Correlation (r)	Median Ratio	IQCD%
$PM_{2.5}$	Approach 2	1.000	1.000	0.300
	Approach 3	1.000	1.030	1.000
PM_{10}	Approach 2	1.000	1.010	0.800
	Approach 3	0.999	1.050	1.600
BC	Approach 2	0.999	1.000	1.700
	Approach 3	0.999	1.020	1.600
UFP	Approach 2	1.000	1.000	0.600
	Approach 3	1.000	1.030	1.200

Note: All comparisons against Approach 1 (full temporal integration). App2 = mean concentration × total ventilation; App3 = integrated concentration × mean ventilation. r = Pearson correlation; IQCD = Interquartile Coefficient of Dispersion of dose ratios.

Table S4. Performance of concentration-based estimators (mean, median, P95, max) versus time-integrated concentration (ATF routes).

Pollutant	Concentration Method	N	r	IQCD%
$PM_{2.5}$	Mean	48	0.995	4.500
	Median	48	0.995	5.400
	P95	48	0.975	9.900
	Max	48	0.942	17.600
PM_{10}	Mean	48	0.993	4.500
	Median	48	0.991	4.400
	P95	48	0.949	15.200
	Max	48	0.643	21.300
BC	Mean	48	0.991	4.300
	Median	48	0.962	12.500
	P95	48	0.950	16.300
	Max	48	0.595	24.800
UFP	Mean	48	0.989	4.500
	Median	48	0.897	7.500
	P95	48	0.924	10.500
	Max	48	0.465	56.400

Note: r = Pearson correlation coefficient; IQCD = Interquartile Coefficient of Dispersion.

Table S5. Performance of concentration-based estimators (mean, median, P95, max) versus inhaled dose (approach 1) (ATF routes).

Pollutant	Concentration Method	N	r	IQCD%
$PM_{2.5}$	Mean	48	0.630	24.000
	Median	48	0.632	22.800
	P95	48	0.592	24.900
	Max	48	0.568	28.300
PM_{10}	Mean	48	0.605	23.200
	Median	48	0.612	24.200
	P95	48	0.540	31.000
	Max	48	0.370	39.800
BC	Mean	48	0.738	24.900
	Median	48	0.705	25.700
	P95	48	0.699	37.700
	Max	48	0.522	36.800
UFP	Mean	48	0.462	25.000
	Median	48	0.214	27.000
	P95	48	0.586	24.000
	Max	48	0.526	52.200

Note: r = Pearson correlation coefficient; IQCD = Interquartile Coefficient of Dispersion.
